# Fighting Consumption in Manchester

**Published:** 1900-02-10

**Authors:** 


### FIGHTING CONSUMPTION IN MANCHESTER.

It is interesting to note the sort of means which an active municipality can take to lessen the dissemination of tuberculosis even without any alteration of existing laws. At a recent meeting of the Manchester Clinical Society Dr. James Niven described what is being done in Manchester in this direction. A constant system of public instruction is maintained, leaflets being distributed year after year to every house in the city, giving instructions as to the precautions which should be taken in the presence of phthisis. The Ladies' Health Society also gives a great amount of instruction in the poorer districts, and in many other ways the public are being educated, although among the poor not much effect has so far been produced. Then the sanitary authority is doing a good deal of disinfection. Voluntary notification of phthisis has now been in operation for some time, so far as the public institutions are concerned. During the past four months 450 notifications have come in, and each case reported is visited, the etiology of the case is investigated as far as possible, precautionary instruction is given, and general disinfection is carried out in the usual manner. In cases in which the patient cannot be shifted from his room the bed-clothes are all changed and disinfected with steam, and the room is disinfected with solution of lime—not chloride of lime. Then fortnightly visits are paid by the visitors of the Ladies' Health Society, who see that strict cleanliness is kept up, and thus, so far as infection is concerned, the rooms and the patients are started afresh, and all that is subsequently necessary is, not successive disinfections, but a sustained cleanliness. What is now wanted is an isolation hospital for the advanced cases, so that when they become bedridden they may be taken out from among the surroundings in the midst of which it is so difficult to maintain conditions inimical to the spread of infection. It is clear that much of the efficacy of what is being done in Manchester hinges upon the constant activity of the Ladies' Health Society, and one cannot but feel that if it is possible by dint of visiting, and urging, and teaching to ensure "a sustained cleanliness" in the homes of the poor, even when their inhabitants are weighted down by sickness, it ought to be possible by the same or some similar agency to ensure in the homes of the healthy also that "sustained cleanliness" which would be one of the most important means of keeping tuberculosis away.

